# High-Efficiency Stem Cell Fusion-Mediated Assay Reveals Sall4 as an Enhancer of Reprogramming

**DOI:** 10.1371/journal.pone.0001955

**Published:** 2008-04-16

**Authors:** Connie C. Wong, Alexandre Gaspar-Maia, Miguel Ramalho-Santos, Renee A. Reijo Pera

**Affiliations:** 1 Department of Obstetrics, Gynecology and Reproductive Sciences, University of California San Francisco, San Francisco, California, United States of America; 2 Institute for Regeneration Medicine, University of California San Francisco, San Francisco, California, United States of America; 3 Diabetes Center, University of California San Francisco, San Francisco, California, United States of America; 4 Doctoral Program in Biomedicine and Experimental Biology, Center for Neuroscience and Cell Biology, University of Coimbra, Coimbra, Portugal; Baylor College of Medicine, United States of America

## Abstract

Several methods allow reprogramming of differentiated somatic cells to embryonic stem cell-like cells. However, the process of reprogramming remains inefficient and the underlying molecular mechanisms are poorly understood. Here, we report the optimization of somatic cell fusion with embryonic stem cells in order to provide an efficient, quantitative assay to screen for factors that facilitate reprogramming. Following optimization, we achieved a reprogramming efficiency 15–590 fold higher than previous protocols. This allowed observation of cellular events during the reprogramming process. Moreover, we demonstrate that overexpression of the Spalt transcription factor, Sall4, which was previously identified as a regulator of embryonic stem cell pluripotency and early mouse development, can enhance reprogramming. The reprogramming activity of Sall4 is independent of an N-terminal domain implicated in recruiting the nucleosome remodeling and deacetylase corepressor complex, a global transcriptional repressor. These results indicate that improvements in reprogramming assays, including fusion assays, may allow the systematic identification and molecular characterization of enhancers of somatic cell reprogramming.

## Introduction

The developmental programs of somatic cells are characterized by remarkably stable patterns of gene expression and repression. Nonetheless, through nuclear reprogramming, the developmental programs of somatic cells may be erased and redirected [1]–[6]. In recent years, much attention has been given to nuclear reprogramming of somatic cells in hopes of generating patient-specific embryonic stem cells (ESCs) that might provide valuable tools for basic science studies and potential novel therapeutics [7], [8].

Nuclear reprogramming was first demonstrated as an integral part of mammalian development; following fusion of the egg and sperm, the fused gametic nucleus must be reprogrammed, through a series of changes that include DNA demethylation and chromatin remodeling, to that of an embryonic cell if development is to be successful [5], [6], [9]. In methods such as somatic cell nuclear transfer (SCNT), the nucleus of a somatic cell is transferred to an enucleated oocyte for reprogramming to an embryonic cell state, through the use of the endogenous machinery [3], [10], [11]. Methods other than SCNT have also been used to reprogram somatic cells including fusion with ESCs and genetic reprogramming via co-expression of pluripotency-associated genes [12]–[16]. Each of these methods has advantages and limitations. For example, although SCNT takes advantage of endogenous programs, it requires the use of oocytes that may be in short supply [17]. In the case of cell fusion, although the cells are in great supply, the procedure results in the formation of tetraploid cells that are genetically unstable [12], [18]–[20]. Finally, although genetic reprogramming by co-expression of the stem cell factors Oct4, Sox2, c-myc and Klf4 is remarkable in that it yields ESCs capable of contributing to both the somatic and germ cell lineages, use of the reprogrammed cells to generate offspring results in increased tumorigenesis in progeny [13]–[16]. Moreover, in all methods, the efficiency of reprogramming is very low, suggesting that additional components of the reprogramming pathways remain to be identified.

In this study, we sought to optimize cell fusion reprogramming protocols, based on fusion of somatic cells and ESCs, in order to screen for enhancers of somatic cell reprogramming. We reasoned that if a factor functions in reprogramming, overexpression of that factor in somatic cells might increase the efficiency with which the cells can be reprogrammed. Thus, we tested whether overexpression of the following factors, individually, increased reprogramming efficiency of MEFs: Oct4, Nanog, Sox2, and Sall4.

## Results

### Optimization of an Efficient Reprogramming Assay

**Figure 1:**
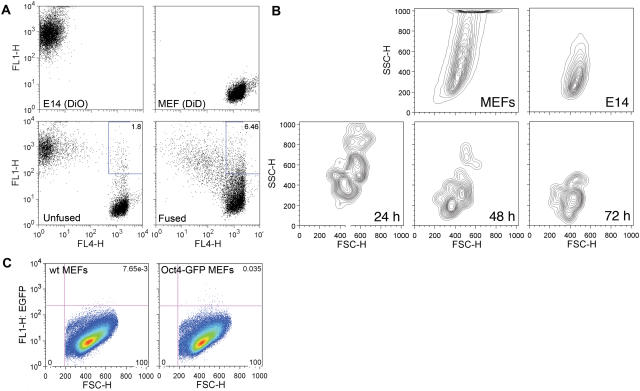
Establishment of an efficient fusion assay. A) Fusion efficiency of the assay. The MEFs and ESCs were stained with the fluorescent dyes Vybrant DiD and Vybrant DiO respectively before fusion. Fusion efficiency was determined by FACS analysis at 5 h post-fusion, using an unfused mixture of cells as a negative control. Note that previous studies have shown that cell surface dyes rarely diffuse across the cell membranes of stained cells [41]. The dual-labeled cells in the unfused population was most likely due to non-specific binding between the ESCs and MEFs. The green fluorescent dye Vybrant DiO was only used in the determination of fusion efficiency but not in a typical reprogramming experiment (due to interference with the observation of GFP signal). B) Morphology change of MEFs during reprogramming. The forward- and side-scatter profiles of the GFP positive cells were FACS analyzed at 24, 48 and 72 h post-fusion. The morphology of the reprogrammed MEFs changed with time to resemble that of the ESCs. C) Quantification of GFP expression. The number of GFP positive cells was FACS analyzed at 24 and 48 h post-fusion (right panel), using wildtype MEFs that did not carry the *Oct4-gfp* transgene but had undergone identical fusion treatment with ESCs as a negative control (left panel). The number of GFP positive cells from wt MEFs was subtracted from that of *Oct4-gfp* MEFs in all calculations.

Several different protocols have been developed to reprogram somatic cells via cell fusion with ESCs, with protocol efficiencies typically less than 0.001% (i.e. ranging from approximately 1 reprogramming event per 1×10^5^ to 4×10^6^ total somatic cells) [12], [20]. Such low efficiencies lead to technical difficulties in screening for positive regulators of somatic cell reprogramming. Thus, we sought to establish an efficient and quantitative reprogramming assay via cell fusion between mouse ESCs and G418-resistant (Rosa26) mouse embryonic fibroblasts (MEFs) that carry the *Oct4-gfp* transgene [20], [21]. We began by exploring conditions required for efficient fusion. Traditionally, cells are fused in suspension in 50% polyethylene glycol [12], [18]–[20]. However, we found that the fusion efficiency was substantially increased by both fusing the ESCs and MEFs in adherent cultures and increasing polyethlyene glycol from 50 to 56%. FACS (fluorescent-activated cell sorting) analysis of MEFs and ESCs, which were fluorescently labeled with Vybrant DiD and Vybrant DiO respectively, indicated that the fusion efficiency was 4.6 +/− 0.1% at 5 h post-fusion (Figure 1A).

The first visible, qualitative evidence of reprogramming (within 24 to 48 h post-fusion) was the expression of the *Oct4-gfp* transgene, which was normally silent in MEFs [22]. As time progressed, the reprogrammed MEFs gradually obtained the morphology of ESCs, as reflected by comparisons of forward and side scatter profiles of the GFP-positive MEFs at 24, 48 and 72 h post-fusion (Figure 1B).

Reprogramming efficiency was quantified by determining: 1) the percentage of cells that expressed *Oct4-gfp* and 2) the number of G418-resistant, stem cell-like colonies formed. The percentage of GFP positive cells was measured by FACS at 24 h and 48 h post-fusion, using wildtype MEFs (without the *Oct4-gfp* transgene) as a negative control (Figure 1C). The number of GFP positive particles from the wt MEFs was subtracted from that of *Oct4-GFP* MEFs in order to eliminate any background fluorescence from our calculations. Typical results indicated that the percentage of GFP positive cells, among the total MEF population at 24 h post-fusion, was 0.029 +/− 0.008% and that the number of reprogrammed colonies obtained by this method was found to be as many as 1 in 6.8×10^3^ total MEF cells, a value 15 to 590 fold higher than previously reported with other reprogramming assays available [12], [18]–[20]. These reprogrammed cells can be expanded in ESC culture conditions, are tetraploid and express markers of pluripotency (data not shown).

### Potential Enhancers of Somatic Reprogramming

**Figure 2:**
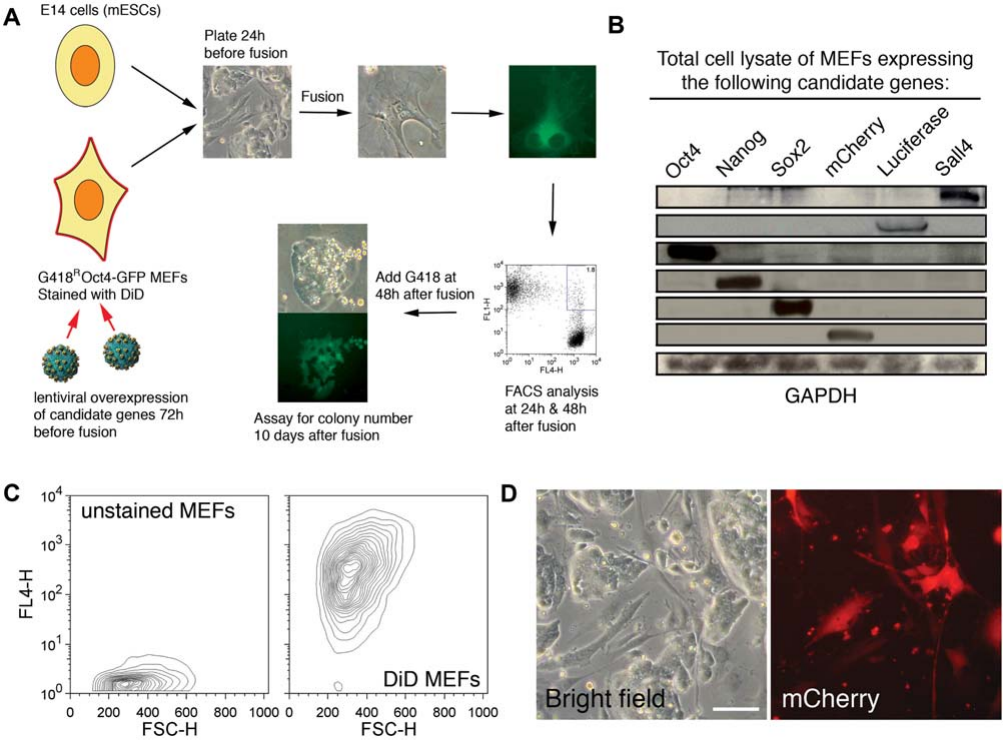
Screen of positive regulators of somatic cell reprogramming. A) Schematic of the screen. Candidate genes were overexpressed in *Oct4-gfp*, G418-resistant MEFs via lentivirus infection 72 h before fusion. B) Successful lentiviral overexpression was verified by Western blotting, as well as expression of the positive control mCherry. C) Infected MEFs were harvested at 24 h before fusion and stained with the fluorescent dye Vybrant DiD. Prepared MEFs were plated with unstained ESCs. GFP expression was FACS analyzed at 24 and 48 h post-fusion. G418 was added to the fused cells at 48 h post-fusion, and the formation of G418-resistant, GFP positive colonies was assayed 10 days post-fusion. D) The visible overexpression of mCherry in infected MEFs indicated the effectiveness of our lentiviral overexpression system. Scale bar represents 50 µm.

The significant improvements in reprogramming efficiency, brought about by the modifications described above, allow for scaling of the assay to multiple-well formats. We next tested whether our protocol was suitable for quantitative analysis of potential reprogramming factors, including those implicated in maintaining pluripotency and in early embryo development: Oct4, Sox2, Nanog and Sall4 [23]–[25]. Previously, Oct4, Sox2, and Nanog have been shown to function in somatic cell reprogramming, whereas the role of Sall4 in this process has not been explored [14], [18]. A schematic of the protocol is shown (Figure 2A).

Aliquots of G418-resistant MEFs, carrying the *Oct4-gfp* transgene were infected with lentivirus constructs that expressed one of the candidate factors 72 h prior to fusion. The overexpression of candidate proteins was confirmed by Western blotting (Figure 2B). 24 h prior to fusion, infected MEFs were harvested and labeled with the fluorescent dye Vybrant DiD (Figure 2C). The fluorescently-labeled MEFs and unstained ESCs were then plated together in triplicate wells (Figure 2D); the visible overexpression of the red fluorescent protein mCherry indicated proper production and infection of the lentiviruses. Cells were harvested at 24 h and 48 h post-fusion, and the percentage of GFP-positive cells among the DiD-positive MEF population was determined. G418 was then added to the remaining well of fused cells 48 h post-fusion and subsequently at 10 days post-fusion, the number of G418-resistant, GFP-positive colonies was determined.

**Figure 3:**
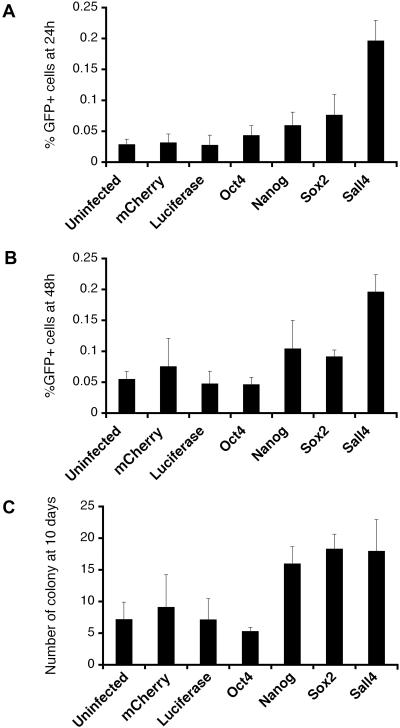
Overexpression of Sall4 enhanced *Oct4-gfp* expression and ES cell-like colony formation in MEFs during reprogramming. The percentage of GFP positive MEFs at: A) 24 h and B) 48 h post-fusion. C) The number of GFP positive, G418-resistant colonies in 1 well of a 6-well plate, 10 days post-fusion. The overexpression of Sall4 positively enhanced both *Oct4-gfp* expression and colony formation of MEFs upon reprogramming.

The onset of *Oct4-gfp* expression provides an initial measure of reprogramming. The percentage of GFP-positive cells, in the population of MEFs that overexpressed each candidate gene, was compared to that of uninfected MEFs and MEFs that overexpressed the negative control proteins, firefly luciferase and the red fluorescent protein, mCherry. At 24 h post-fusion, GFP expression in MEFs that overexpressed negative control proteins was similar to that of the uninfected control, indicating that lentiviral-mediated protein overexpression did not affect GFP expression in MEFs carrying the *Oct4-gfp* transgene (Figure 3A). Unexpectedly, however, MEFs that overexpressed the known reprogramming facilitators, Oct4, Nanog and Sox2, also did not show a significant increase in *Oct4-gfp* expression relative to controls. Although this was unexpected, the lack of enhanced GFP expression when Oct4, Nanog and Sox2 were overexpressed in MEFs might be attributed to several possibilities (this is further described in the discussion section below). In contrast, we observed that the percentage of GFP-positive cells in MEFs that overexpressed the Spalt transcription factor, Sall4, increased 7-fold relative to controls. The comparison of *Oct4-gfp* expression at 48 h post-fusion was similar to that at 24 h (Figure 3B). MEFs that overexpressed Sall4 consistently demonstrated the highest percentage of GFP-positive cells compared to the other candidate genes.

A second measure of reprogramming is colony formation. With G418 drug selection, GFP-positive colonies began to appear within 5 days post-fusion. The total number of colonies was recorded 10 days post-fusion (Figure 3C). The number of reprogrammed colonies formed by MEFs overexpressing negative control proteins was again similar to the uninfected control, confirming that the lentivirus itself did not affect reprogramming. Nanog, Sox2 and Sall4 all showed a significant increase in the number of reprogrammed colonies relative to controls (*p*<0.05). However, the overexpression of Oct4 in MEFs did not promote formation of reprogrammed colonies in these assays (Figure 3C).

### Confirmation of Sall4 as an Enhancer of Reprogramming by Cell Fusion

**Figure 4:**
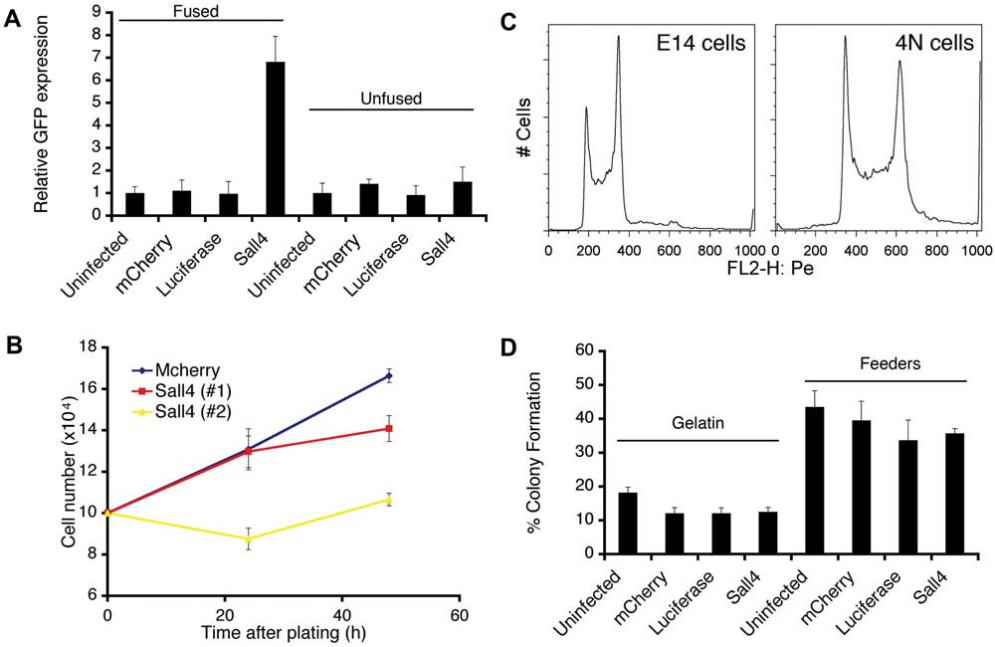
Sall4 is a *bona fide* positive regulator of reprogramming. A) Overexpression of Sall4 in *Oct4-gfp* MEFs did not induce GFP expression. Sall4, mCherry and luciferase were overexpressed in *Oct4-gfp* MEFs via lentivirus infection. Half of the infected MEFs was fused to ESCs as described, while the other half was not. Only MEFs overexpressing Sall4 and fused to ESCs showed an increased number of GFP positive cells when compared to the negative controls, indicating that overexpression of Sall4 alone did not induce GFP expression. The numbers of GFP positive cells in the infected cells relative to that of the uninfected cells were shown. B) Overexpression of Sall4 did not increase cell doubling time in MEFs. mCherry and two different constructs of Sall4 were overexpressed in MEFs, which were plated onto 6 well plates and assayed for cell number every 24 h. Note that another clone of Sall4 of identical sequence was used as a duplicate. C, D) Infection of Sall4-expressing lentiviruses did not increase the colony formation efficiency in ESCs. Both ESCs and previously reprogrammed MEFs that were tetraploid were infected with lentiviruses expressing Sall4, mCherry or luciferase. D) The infected cells were plated either on gelatin or on feeder cells. The number of colonies formed was assayed after 7 days.

As described above, results indicated that Sall4 was likely a positive regulator of somatic cell reprogramming, contributing to both early activation of *Oct4* in the somatic cells and formation of reprogrammed colonies. However, given this data, we also considered whether Sall4 might directly activate the *Oct4-gfp* transgene in the absence of overall reprogramming. To examine this alternative possibility, we tested whether increased GFP expression at 24 h and 48 h post-fusion was due to transcription activity of Sall4 alone. For this purpose, we overexpressed Sall4, as well as the negative controls, in MEFs carrying the *Oct4-gfp* transgene. Half of the infected MEFs were then cultured alone, and the remainder was fused with ESCs. Then, when the 24 h time point would typically be analyzed in a fusion experiment, cells were harvested and the percentage of GFP-positive cells was determined (Figure 4A). We found that the observed increase in the number of GFP-positive cells was dependent on fusion with ESCs; MEFs that overexpressed Sall4 but were not fused with ESCs did not demonstrate increased *Oct4-gfp* expression. This indicated that increased GFP expression in cells overexpressing Sall4 is not a direct effect of Sall4 interacting with the *Oct4* promoter of the *Oct4-gfp* transgene, but rather is a result of the enhancement of reprogramming.

Next, we tested whether overexpression of Sall4 altered the growth rate of MEFs, thus leading to an increased number of colonies unrelated to reprogramming. For this purpose, we overexpressed the negative control, mCherry, and Sall4 in MEFs, plated the cells and determined cell number every 24 h as shown (Figure 4B). An independent clone of Sall4 of identical sequence was used in this experiment as a duplicate; clone 1 was the construct used in all other experiments described in this study. Results indicated that overexpression of Sall4 did not increase, but instead slightly decreased, the growth rate of MEFs relative to the control.

We also addressed whether expression of Sall4 enhanced plating efficiency of ESCs. For this purpose, we used both wildtype ESCs and subcloned lines of tetraploid (4N, Figure 4C) reprogrammed cells. In order to test the effect of Sall4 overexpression on ESC colony formation efficiency, we infected ESCs and 4N cells with constructs that expressed the negative control proteins and Sall4. We observed under a microscope that the fluorescent intensity of the negative control, mCherry, was significantly lower in infected ESCs than in infected MEFs from previous experiments; Western blotting also suggested that the expression level of Sall4 in infected ESCs was not significantly increased relative to endogenous levels (data not shown). This may reflect different activity, or susceptibility to silencing, of the CMV promoter in MEFs relative to ESCs [26]. Nonetheless, we reasoned that the lower expression levels in ESCs parallel observations during reprogramming: when reprogrammed mCherry-infected MEFs gain ESC-like characteristics after fusion, there appears to be a sharp decline of mCherry expression during colony formation (data not shown). After infection, we plated the infected ESCs and 4N cells with and without feeders, and assayed colony formation after 7 days (Figure 4D). The efficiency of forming colonies in uninfected ESCs, or ESCs infected with constructs expressing the negative controls mCherry and luciferase, was approximately 20% on gelatin and 45% on feeder cells, similar to previously reported values [27]. We observed that cells infected with the Sall4 construct also did not demonstrate enhanced ability to form colonies in either the presence or absence of feeders. Similar results where obtained with expression of Sall4 in the 4N reprogrammed ESCs (data not shown).

### Structure-Function Studies of Reprogramming by Cell Fusion

Taken together, the data described above suggested that Sall4 is a positive regulator of somatic reprogramming. Sall4 is a zinc finger transcription factor expressed in cells of the early embryo and the germ line, and is required for maintenance of pluripotency [28]–[30]. Sall4 may act as both a positive transcriptional regulator of genes such as *Oct4* [29] and as a transcriptional repressor [31]. The Sall family of proteins contains an N-terminal 12-amino acid motif that recruits the nucleosome remodeling and deacetylase corepressor (NuRD) complex, which is involved in global transcriptional repression and regulation of specific developmental processes [31], [32]. The C-terminal region of Sall4 has also been shown recently to contain weak transcription repression activity as well [33].

**Figure 5:**
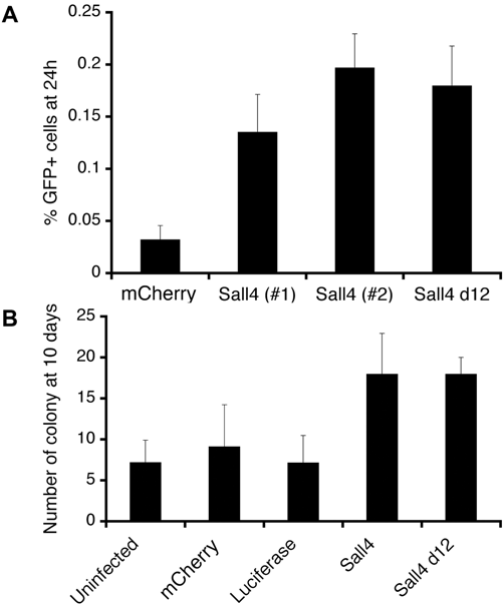
The N-terminal domain is not required for Sall4 function in reprogramming. A) Sall4 d12 mutant behaved similarly to wt Sall4 in reprogramming. Sall4 d12, as well as two clones of wt Sall4 of identical sequences, were overexpressed in *Oct4-gfp* MEFs, which were then fused to ESCs and assayed for GFP expression as described. B) The overexpression of Sall4 d12 resulted in a similar increase in the number of *Oct4-gfp* cells as wt Sall4, as well as a similar increase in the number of reprogrammed colonies.

We sought to determine if our quantitative protocol for reprogramming could be used to dissect the structure-function relationships of factors implicated in reprogramming, such as Sall4. Thus, we tested whether the N-terminal 12-amino acid motif of Sall4 is required for somatic cell reprogramming. For this purpose, we generated a truncated Sall4 mutant (Sall4 d12) that lacked the N-terminal 12-amino acid motif and repeated the fusion assays (Figure S1). We found that overexpression of Sall4 d12 resulted in both early activation of *Oct4-gfp* (Figure 5A) and in increased numbers of ESC-like colonies (Figure 5B), similar to results with wildtype Sall4. We also noted that overexpression of Sall4 d12 did not alter the GFP expression pattern or growth rate of MEFs carrying the *Oct4-gfp* transgene, nor did overexpression increase colony formation efficiency (Figure S2, S3, S4). These data show that the enhancement of somatic cell reprogramming by Sall4 does not require the N-terminal domain of the protein that has been implicated in recruiting the NuRD complex. Furthermore, these data suggest that improvements in the fusion assay may provide a useful platform for future structure-function studies of regulators of reprogramming.

## Discussion

In this study, we optimized the cell fusion reprogramming assay. The assay makes use of G418-resistant, *Oct4-gfp* MEFs and mouse ESCs. Due to improved fusion and reprogramming efficiencies, the assay is now potentially amenable to screening formats as demonstrated here with the analysis of overexpression of the pluripotency factors Oct4, Nanog, Sox2 and Sall4. Moreover, by taking advantage of the fact that the *Oct4-gfp* transgene is activated within the first 24–48 h of reprogramming, the assay allows for further physical and molecular characterization of the reprogramming process by microscopy and FACS.

### Sall4: An Enhancer of Reprogramming

In this study, we demonstrate that the transcription factor Sall4 can enhance somatic cell reprogramming as evidenced by both enhanced *Oct4-gfp* expression and colony formation. Previously, Sall4 had not been shown to function in somatic cell reprogramming. Sall4 is a member of the Spalt family of transcription factors which was originally identified in *Drosophila* as a homeotic gene required for head and tail development [28], [34], [35]. In mammals, Sall4 is essential for early embryo development including establishment and maintenance of the early cell lineages of the inner cell mass [30]. Sall4 is also essential for the maintenance of pluripotency and self-renewal of ESCs and for their derivation from blastocysts [30]. Although Sall4 may act as a transcription factor that regulates numerous genes, one of the few known target genes is *Oct4* [29]. Recent studies show that Sall4 interacts with Nanog to control the expression of Oct4 [36]. Together, Oct4, Nanog, Sox2 and Sall4 form a regulatory circuit to maintain pluripotency of ESCs, prompting our exploration of these factors [36]–[38]. Our results suggesting that Sall4 enhances reprogramming in cell fusion prompts further analyses regarding whether it may enhance reprogramming in other reprogramming strategies; in addition, it is very likely that additional enhancers remain to be identified.

### Comparisons to Other Reprogramming Assays

A previous report by Silva and colleagues demonstrated that overexpression of Nanog in mouse ESCs enhances reprogramming of neural stem cells nearly 200-fold and reprogramming of MEFs 10-fold as measured by colony formation [18]. In the current study, when we overexpressed Nanog in MEFs rather than in ESCs, surprisingly, we achieved only a 3-fold increase in reprogramming efficiency as judged by colony formation. Further consideration and comparison of these studies is merited: First, it is apparent from several studies, including that of Silva and colleagues, that it is more difficult to reprogram somatic cells such as MEFs than neural stem cells [18], perhaps due to the state of differentiation of MEFs and/or epigenetic status of key pluripotency genes. Second, we note that overexpression of Nanog in ESCs resulted in greater enhancement of reprogramming efficiency compared to overexpression in MEFs. This observation might reflect fundamental differences between the two studies. Since Nanog is an important pluripotency factor, it is highly likely that the overexpression of Nanog in ESCs may reinforce the pluripotency regulatory circuit, or stem cell properties, of ESCs. In contrast, in our study, the overexpression of Nanog and other positive regulators of reprogramming in MEFs most likely enhances reprogramming by priming and preparing the somatic cell genome for reprogramming. Thus, we suspect, from comparisons of this data to that from other publications, that the latter is a far less efficient process than reinforcing the pluripotency regulatory circuit of ESCs.

Recently, several reports have demonstrated that MEFs can be reprogrammed by co-overexpressing the pluripotency factors Oct4, Sox2, C-myc and Klf4 [13]–[15]. In our study, neither Oct4 nor Sox2 overexpression in the somatic compartment led to early activation of *Oct4* during reprogramming of MEFs, and only Sox2 led to increased numbers of reprogrammed colonies. It is possible that activation of *Oct4* is not one of the earliest events to occur during reprogramming, and clearly that not all factors that facilitate reprogramming will lead to early activation of *Oct4*. Thus, the lack of early Oct4 activation in our assay does not preclude a factor from being an enhancer of reprogramming. Furthermore, the expression level of Oct4 is regulated in a precise manner in ESCs such that an increase in Oct4 expression level leads to differentiation into primitive endoderm and mesoderm, whereas a decrease results in trophectoderm formation [39]. Thus, we speculate that overexpression of Oct4 alone without other reprogramming factors may actually inhibit reprogramming.

### Conclusions

Finally we note that it is possible that the role of reprogramming factors may differ depending on the method of reprogramming, be it SCNT, cell fusion or over-expression of a subset of genetic factors. The fusion reprogramming assay as optimized here is useful for identification and characterization of new regulators or enhancers of somatic reprogramming and may bypass some of the difficulties with other methods. Together, the array of methods for reprogramming holds great promise for the generation of patient-specific stem cells for use in diverse basic and clinical studies in the future.

## Materials and Methods

### Cell Culture

Mouse ESCs (E14) were cultured on plates coated with 0.1% gelatin (Sigma-Aldrich, Steinheim, Germany) in ESC medium [(Dulbecco's modified Eagle's medium (DMEM) (Invitrogen, Carlsbad, CA) supplemented with 15% knockout serum replacement (Invitrogen), 2 mM L-glutamine (Invitrogen), 1 mM sodium pyruvate (Invitrogen), 1% non-essential amino acids (Invitrogen), 0.57 µM beta-mercaptoethanol (Sigma-Aldrich), 1% penicillin/streptomycin (Invitrogen), and 0.3% leukemia inhibitory factor]. MEFs were harvested from (*Rosa26 X Oct4-gfp*) transgenic mice as described [20], [21] and cultured on gelatinized plates in MEF medium [DMEM supplemented with 10% fetal bovine serum (Hyclone Labs, Logan, UT), 2 mM L-glutamine, 1 mM sodium pyruvate, 1% non-essential amino acids and 1% penicillin/streptomycin]. 293 cells for lentivirus production were cultured on gelatinized plates in MEF medium.

### Lentiviral Vectors and Overexpression

The lentivirus overexpression vector pLove has been described [40]. The candidate genes were cloned individually into pEntr-1A (Invitrogen), then subcloned into pLove using the Gateway® technology (Invitrogen) according to the manufacturer's protocol. The companion vectors for lentivirus production, pMDL, pRSV and pVSV-G, were gifts from Dr. Michael McManus (University of California, San Francisco, CA).

The 293 cells were plated on 15-cm plates at 80000 cells/cm^2^ 12–24 h before transfection. 4 µg of pLove and 1.3 µg each of pMDL, pRSV and pVSV-G were transfected into 293 cells with FuGENE 6 transfection reagent (Roche Applied Science, Indianapolis, IN) according to the manufacturer's protocol. Supernatant containing mature lentivirus was harvested at 48 h to 72 h after transfection and filtered with 0.45 µm PVDF syringe filters (Millipore, Billerica, MA). For infection, 10 ml of the filtered supernatant and 5 ml of fresh MEF medium was added to MEFs cultured in 10-cm plates for 24 h. The cells were then rinsed thoroughly 3× with DMEM and continued to culture in fresh MEF medium for another 24 h. Overexpression of candidate genes was verified by Western blotting. MEFs overexpressing the candidate genes were harvested and homogenized in RIPA buffer [50 mM Tris, 150 mM NaCl, 0.5% sodium deoxycholate, 1% NP-40, 0.1% SDS, pH 8.0] at 100000 cells/µl. After a clarifying centrifugation step at 12000 rpm for 20 min at 4°C, 30 µl of 6× Lammeli buffer [0.3 M Tris pH 6.8, 36% glycerol, 10% SDS, 120 µg/ml bromophenol blue] and 2 µl of betamercaptoethanol were added to 60 µl of cell lysate, of which 20 µl was loaded per lane on a 10% SDS-polyacrylamid gel. Western blotting was performed using a goat anti-V5 antibody (Abcam, Cambridge, MA) to detect the expression of all V5-tagged candidate genes, and a goad anti-GAPDH antibody (Abcam) to detect the expression of GAPDH as a loading control.

### Cell Fusion Assay

At 24 h before fusion, G418-resistant, *Oct4-gfp* MEFs overexpressing the candidate genes were stained with 0.5 µl/ml Vybrant DiD (Invitrogen) in DMEM for 20 min at 37°C. The cells were thoroughly rinsed 3× with phosphate buffered saline (PBS) before trypsinized and replated on 6-well plates with unstained ESCs; both MEFs and ESCs were seeded at 3×10^5^ cells per well in ESC medium. During fusion, the cells were first rinsed 1× with 2 ml PBS (pH 7.4) per well, then primed with 1 ml 50 µM sodium dodecyl sulphate (Sigma-Aldrich) in PBS for 3 min at 37°C before incubating with 1 ml 56% PEG-3350 (Sigma-Aldrich) resuspended in PBS for 1 min at 37°C [41]. DMEM was then added to the wells at 1 ml/min to dilute the PEG solution for up to 5 ml. The cells were rinsed 1× with 2 ml DMEM, 1× with 2 ml ESC medium before returning to 3 ml ESC medium. The medium was fully replaced daily post-fusion. At 24 h and 48 h post-fusion, the fused cells were harvested and resuspended in PBS-1% bovine serum albumin (Sigma-Aldrich) before assaying for GFP expression with a FACSCalibur (BD Biosciences, San Jose, CA). 200 µg/ml G418 solution (Invitrogen) was added at 48 h post-fusion to begin the selection for reprogrammed colonies. In order to control for background fluorescence in our FACS analysis, we fused both wt MEFs that did not contain any *gfp* transgene, and MEFs that carried the *Oct4-gfp* transgene to ESCs independently in our fusion experiments. We measured the number of GFP positive cells in both populations, and we subtracted the number of GFP positive particles of the wt MEFs from that of *Oct4-gfp* MEFs in order to eliminate background fluorescence from our calculations. All fusion experiments were repeated between 3–6 times. The data were then pooled and the average and standard deviation were calculated. Post-hoc tests following a univariate analysis of variance (ANOVA) show that average number of colony for Nanog, Sall4, and Sox2 are significantly different from those of the uninfected, luciferase and mCherry controls (*p*<0.05).

For the analysis of fusion efficiency described in Figure 1A, MEFs were stained with 0.5 µl/ml Vybrant DiD (Invitrogen) and ESCs were stained with 0.5 µl/ml Vybrant DiO (Invitrogen) in DMEM for 20 min at 37°C before cell fusion. Cells were allowed to recover in ESC medium for 5 h before FACS analysis.

## Supporting Information

Figure S1 Sall4 d12 overexpression. Overexpression of Sall4 d12 in Oct4-GFP MEFs was verified via Western blotting using antibodies against Sall4 (gifts from Dr. Huck-Hui Ng from Nanyang Technological University, Singapore). Sall4 was expressed in wildtype mouse ESCs but not uninfected MEFs.
(0.11 MB TIF)

Figure S2 Overexpression of Sall4 d12 in Oct4-gfp MEFs did not induce GFP expression. Sall4 d12 was overexpressed in Oct4-gfp MEFs and the activation of Oct4-GFP was measured as described in the main text and Figure 4A.
(0.21 MB TIF)

Figure S3 Overexpression of Sall4 d12 did not increase cell doubling time in MEFs. Sall4 d12 was overexpressed in Oct4-gfp MEFs and the doubling time of MEFs was measured as described in the main text and Figure 4B.
(0.26 MB TIF)

Figure S4 Overexpression of Sall4 d12 did not increase the colony formation efficiency in MEFs. Sall4 d12 was overexpressed in E14 and the tetraploid reprogrammed MEFs, and the colony forming efficiency of the infected ESCs was measured as described in the main text and Figure 4D.
(0.21 MB TIF)
