# Supplementary figures and images for: High-Efficiency Stem Cell Fusion-Mediated Assay Reveals Sall4 as an Enhancer of Reprogramming
Source: PLoS One. 2008 Apr 16;3(4):e1955. doi: 10.1371/journal.pone.0001955 (PMC2278370; doi:10.1371/journal.pone.0001955)

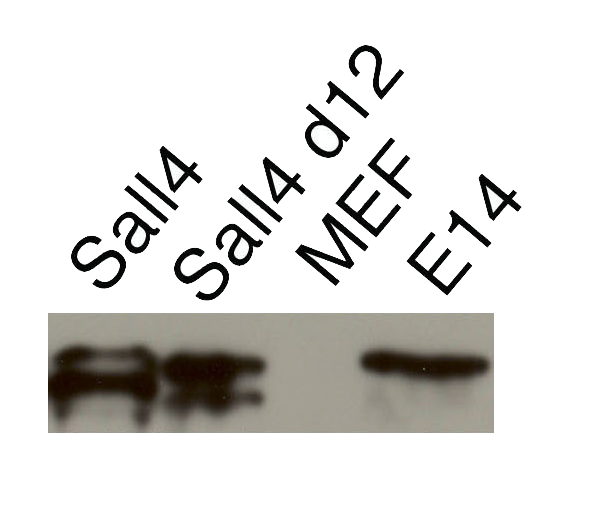

Supplement: Figure S1 — Sall4 d12 overexpression. Overexpression of Sall4 d12 in Oct4-GFP MEFs was verified via Western blotting using antibodies against Sall4 (gifts from Dr. Huck-Hui Ng from Nanyang Technological University, Singapore). Sall4 was expressed in wildtype mouse ESCs but not uninfected MEFs. (0.11 MB TIF) [file pone.0001955.s001.tif]

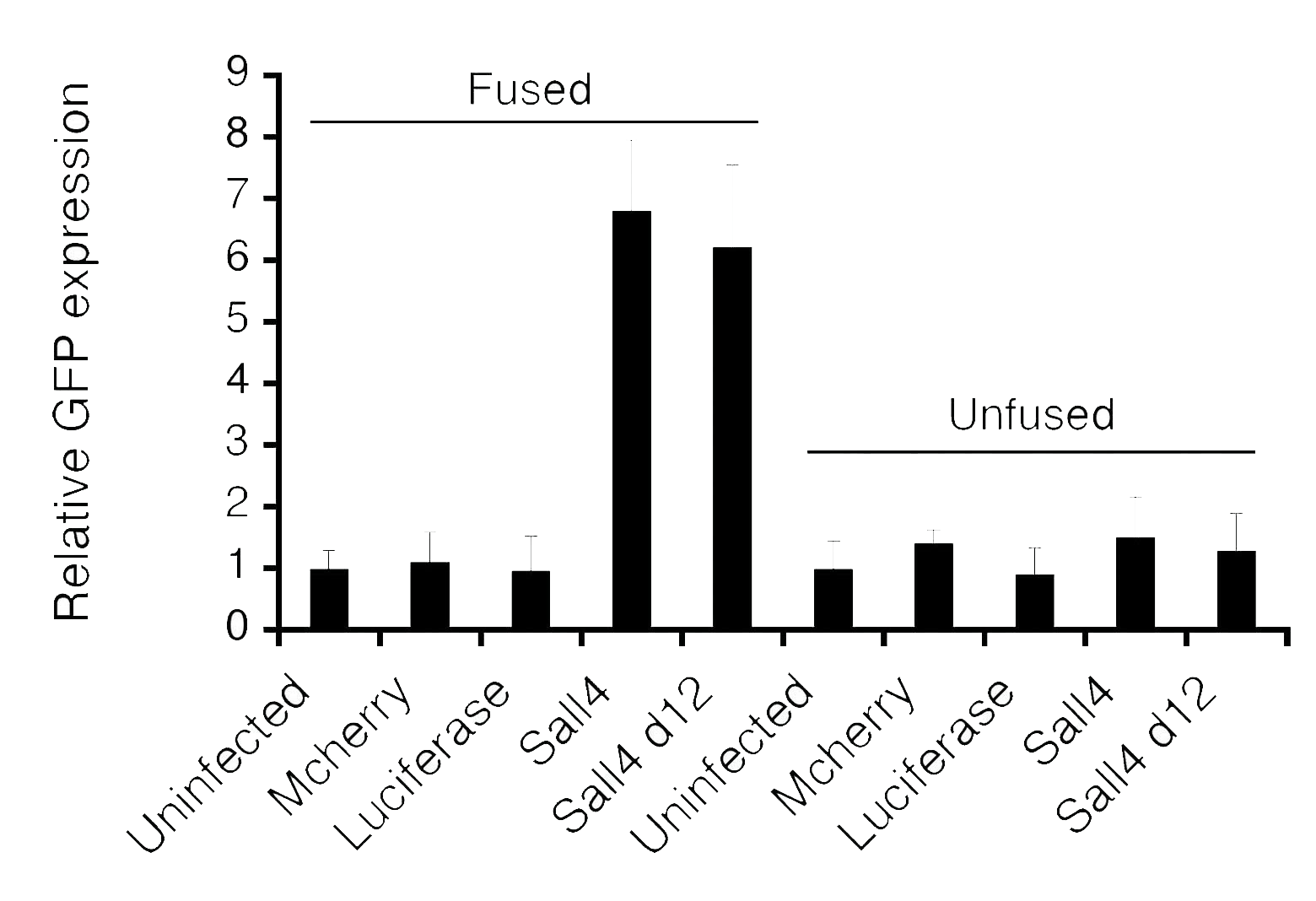

Supplement: Figure S2 — Overexpression of Sall4 d12 in Oct4-gfp MEFs did not induce GFP expression. Sall4 d12 was overexpressed in Oct4-gfp MEFs and the activation of Oct4-GFP was measured as described in the main text and Figure 4A. (0.21 MB TIF) [file pone.0001955.s002.tif]

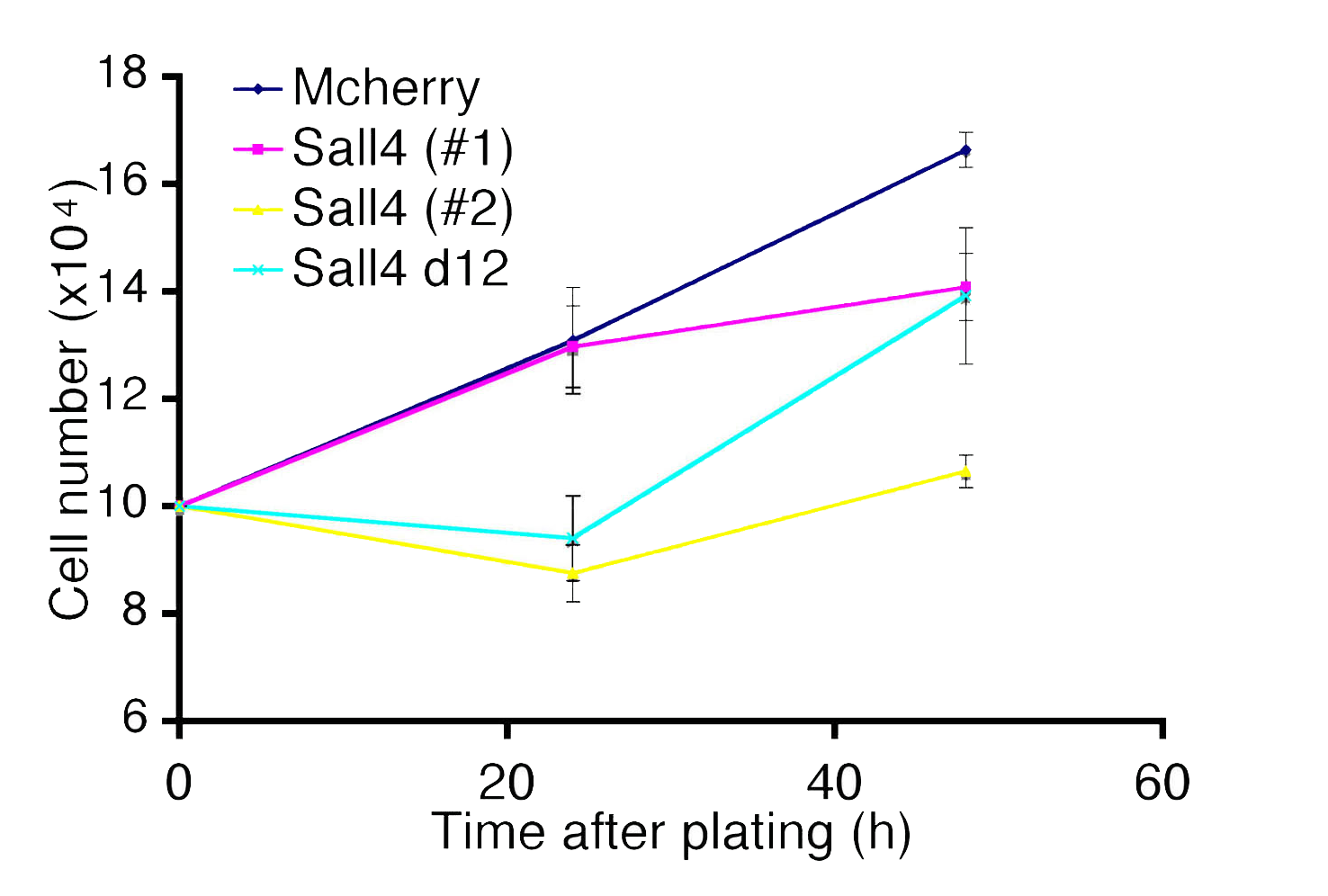

Supplement: Figure S3 — Overexpression of Sall4 d12 did not increase cell doubling time in MEFs. Sall4 d12 was overexpressed in Oct4-gfp MEFs and the doubling time of MEFs was measured as described in the main text and Figure 4B. (0.26 MB TIF) [file pone.0001955.s003.tif]

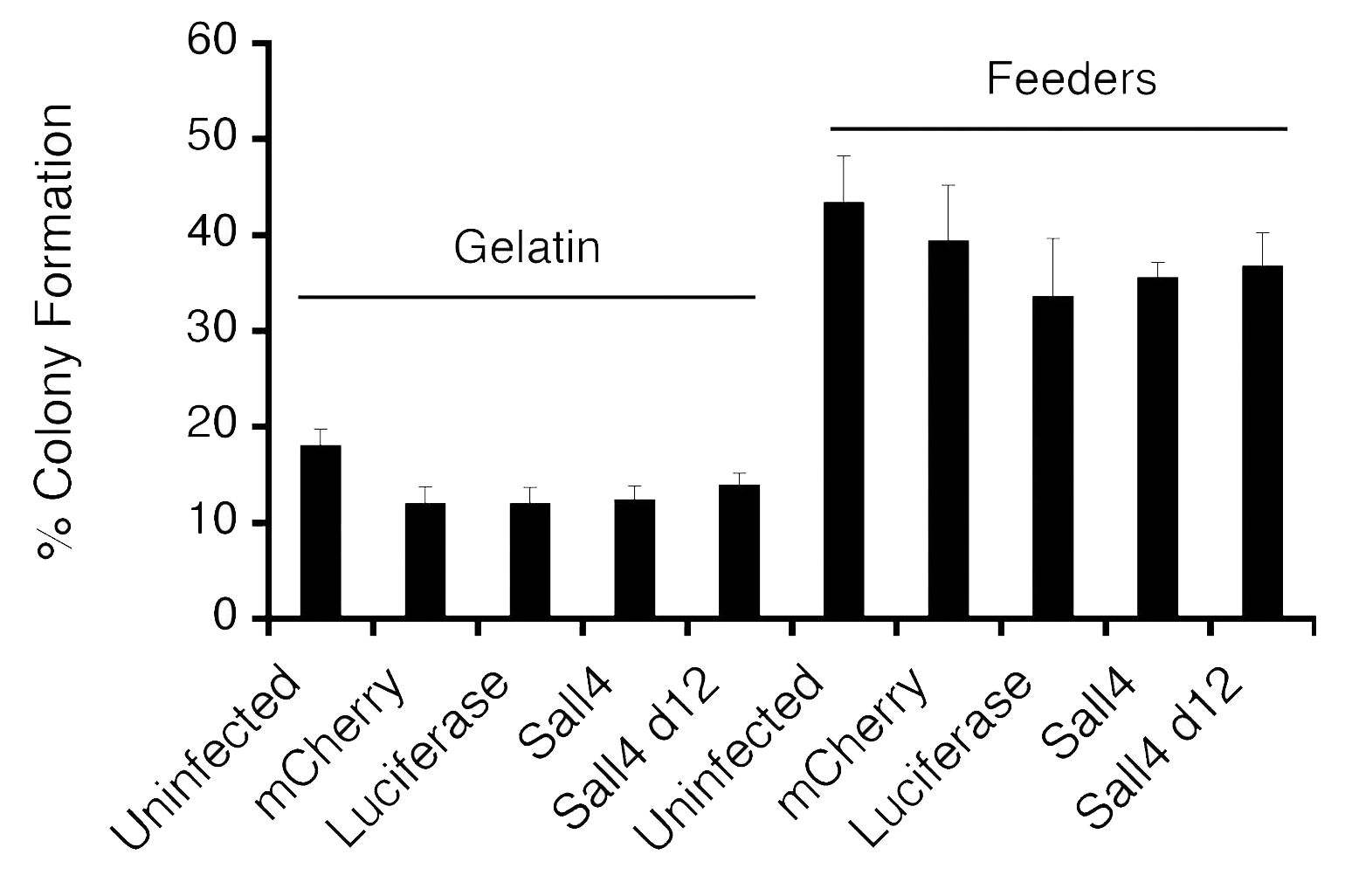

Supplement: Figure S4 — Overexpression of Sall4 d12 did not increase the colony formation efficiency in MEFs. Sall4 d12 was overexpressed in E14 and the tetraploid reprogrammed MEFs, and the colony forming efficiency of the infected ESCs was measured as described in the main text and Figure 4D. (0.21 MB TIF) [file pone.0001955.s004.tif]
